# Supplementary material for: Unsupervised clustering and epigenetic classification of single cells
Source: Nat Commun. 2018 Jun 20;9:2410. doi: 10.1038/s41467-018-04629-3 (PMC6010417; doi:10.1038/s41467-018-04629-3)
Supplement: Supplementary file 1 — Supplementary Information [file 41467_2018_4629_MOESM1_ESM.pdf]

Unsupervised clustering and epigenetic classification of single cells

Zamanighomi *et al.*

Supplementary Information

Contents

1 Supplementary Figures **2**

2 Supplementary Tables **21**

3 Supplementary Note **26**
3.1 Data processing 26
3.2 Choosing the number of clusters 27
3.3 Computation of the maximum likelihood estimate of regression coefficient 27
3.4 Computation of the maximum a posteriori estimate 28
3.5 Computation of the standard error 28
3.6 Experimental mixtures of single cells 28
3.7 The evaluation of cluster specific peaks 29
3.8 The evolution of acute myeloid leukemia 29

1 Supplementary Figures

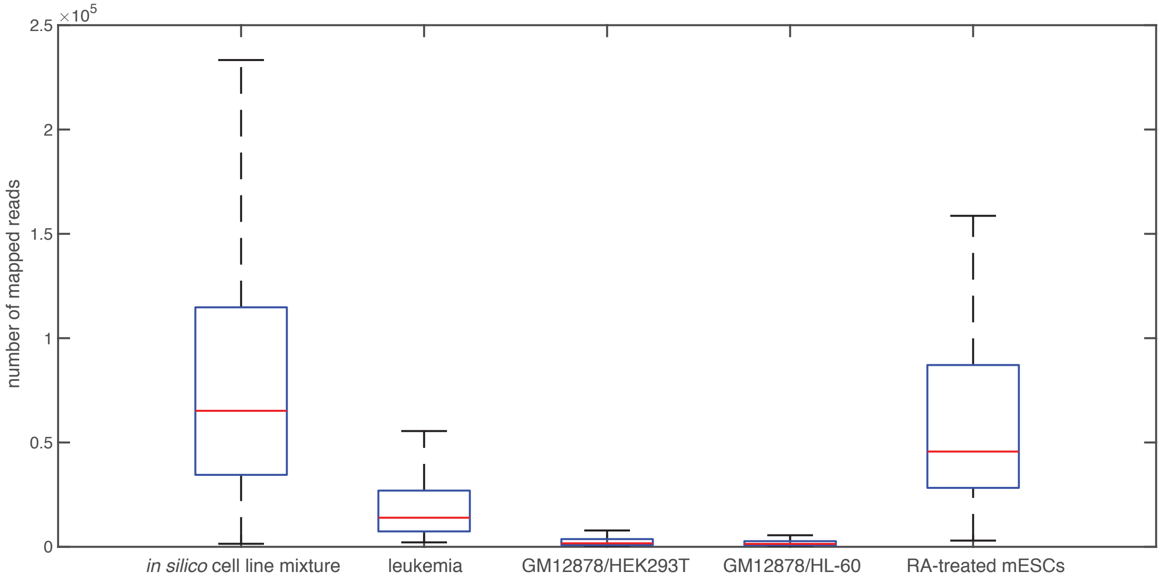

Supplementary Figure 1: **Comparison of read counts for scATAC-seq datasets.** Boxplots of read counts mapped to hg19 for cells in the *in silico* cell line, leukemia, GM12878/HEK293T, and GM12878/HL-60 mixtures. We also depict the box plot of read counts mapped to mm9 for RA-treated mESCs. The red line indicates the median and the blue box gives the interquartile range, plotted using the default boxplot function in MATLAB. The dashed lines extend to 1.5 times the interquartile range.

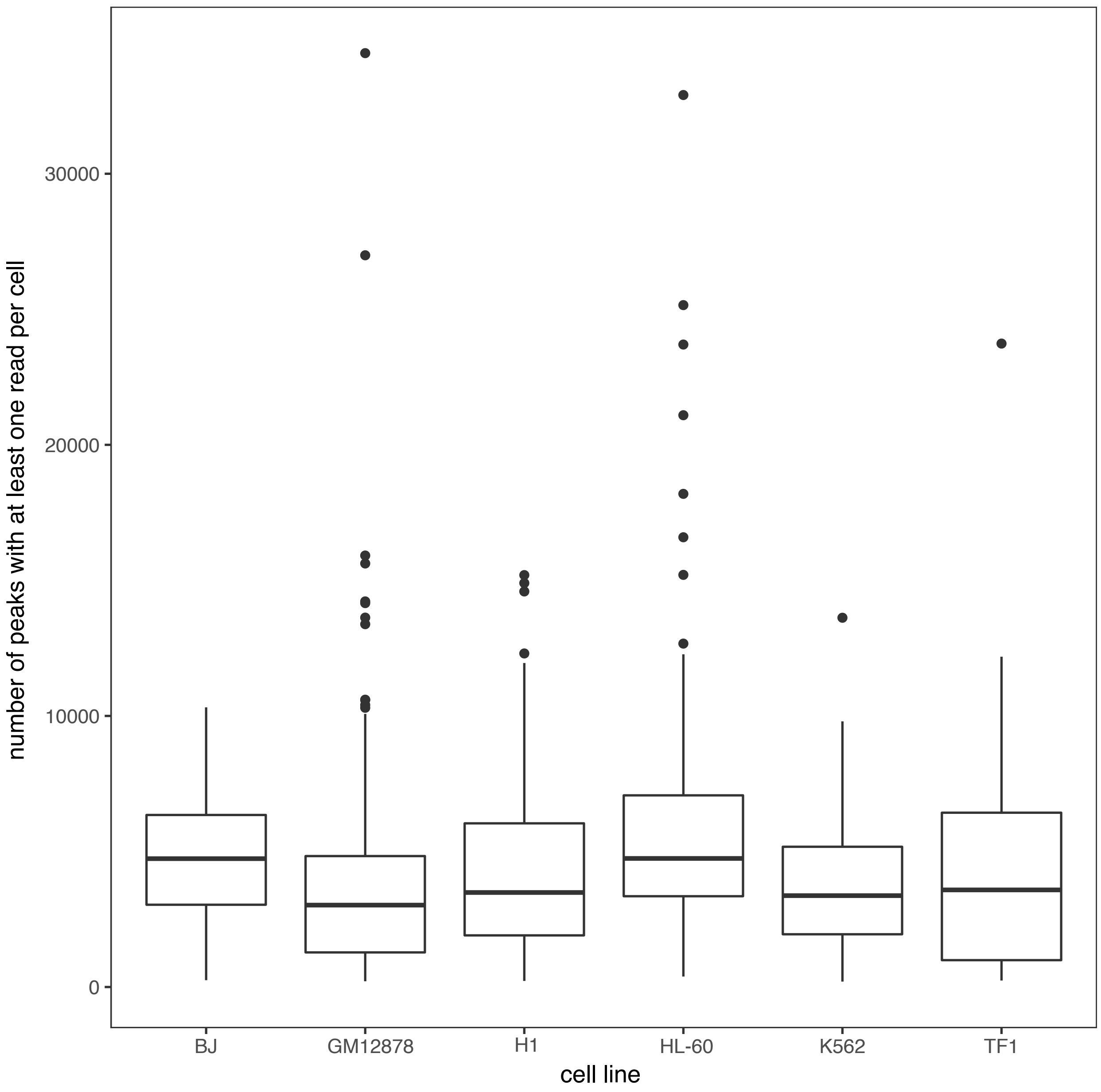

Supplementary Figure 2: **Comparison of the number of peaks with non-zero counts per cell for scATAC-seq datasets**. Boxplots of the number of peaks with at least one overlapping read per cell for the *in silico* mixture, plotted using the default boxplot function in R. The solid line is the median, the box is the interquartile range and the lines extend to the extrema or to 1.5 times the interquartile range.

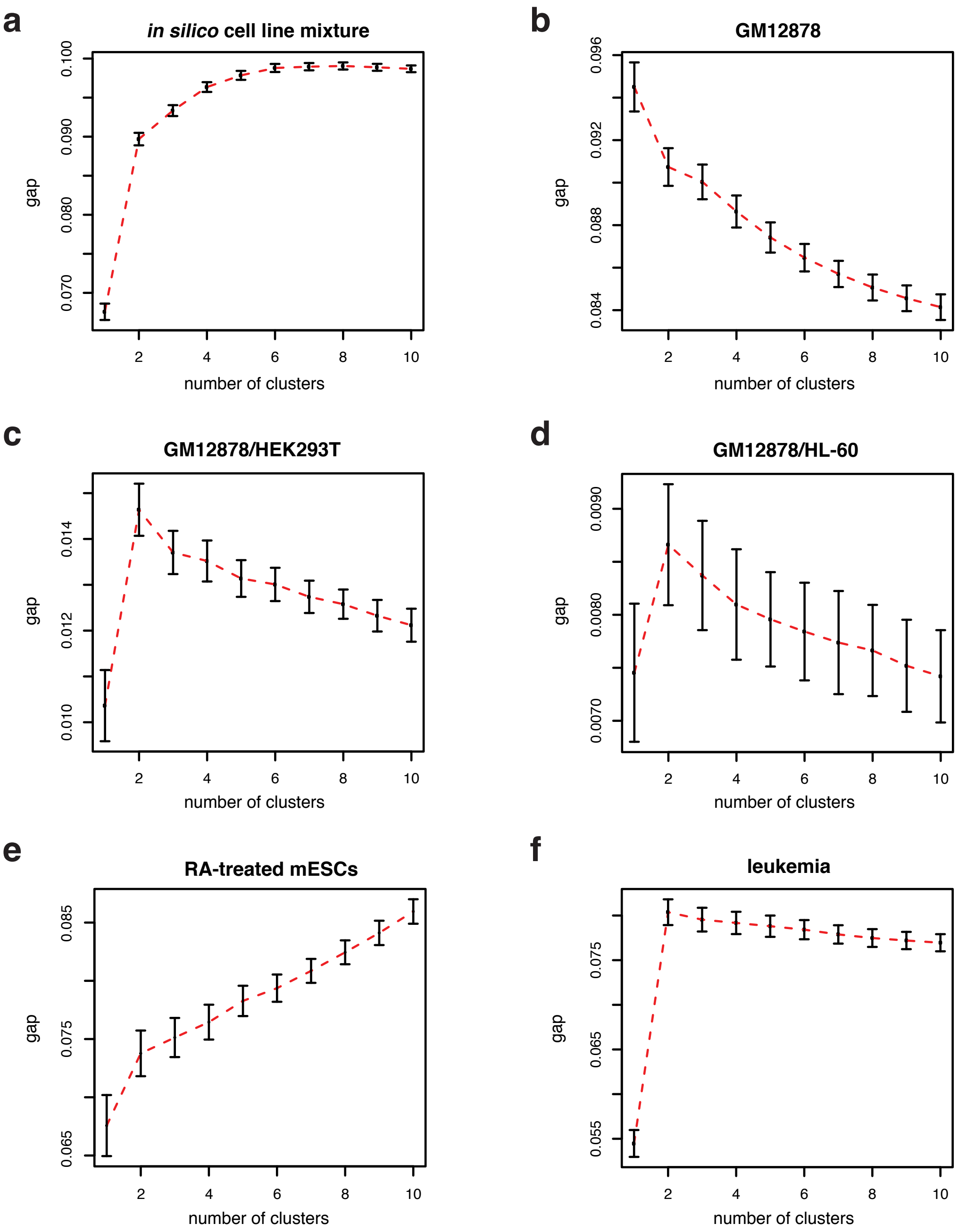

Supplementary Figure 3: **Gap statistic plots for *scABC*.** (**a**) For the *in silico* mixture of six cell lines, the selected number of clusters $\hat{K} = 6$. (**b**) For the GM12878 cell line, the selected number of clusters $\hat{K} = 1$. (**c**) For the mixture of GM12878/HEK293T cell lines, the selected number of clusters $\hat{K} = 2$. (**d**) For the mixture of GM12878/HL-60 cell lines, the selected number of clusters $\hat{K} = 2$. (**e**) For the RA-treated mESCs, the selected number of clusters $\hat{K} = 2$. (**f**) For the leukemia mixture, the selected number of clusters $\hat{K} = 2$. Error bars represent the standard deviation for $B = 10$ simulated reference data sets. Details for the calculation of $\hat{K}$ is presented in Supplementary Notes.

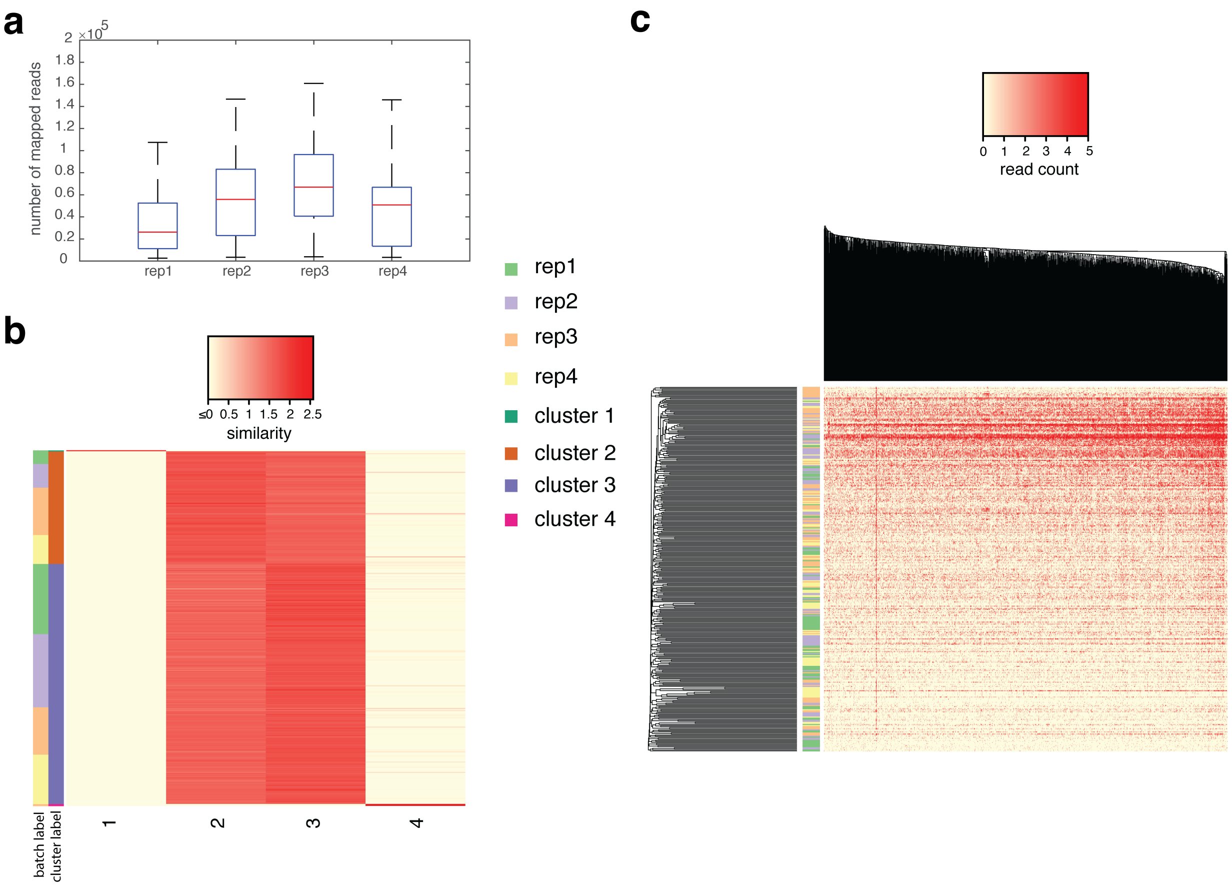

Supplementary Figure 4: ***scABC* is robust to batch effects.** (**a**) Similar to Supplementary Figure 1, we depict the box plot of read counts mapped to hg19 for cells in each GM12878 batch: rep1, rep2, rep3, and rep4. (**b**) Similarity between individual cells (rows) and the four landmarks defined by *scABC* (columns), with the corresponding cluster and batch assignments shown on the left. Notably, *scABC* determined that the total number of clusters K is equal to one (Supplementary Fig. 3), however, we set $K = 4$ to further investigate the robustness of our method to batch effects. Similarity is defined as the Spearman correlations of cells and landmarks (details in Method). For a better comparison of landmarks, throughout figures, we also normalize the correlations between each cell and landmarks using the mean of their absolute values. The majority of cells are split into two similar clusters that are not specific to any replicates, suggesting that *scABC* is not affected by batch biases. Although clusters 2 and 3 are quite analogous, we observe small variations between these two clusters that could indicate the heterogeneity of GM12878 cell line (see Supplementary Fig. 12 for detailed analysis). (**c**) Heat map for the read counts across peaks and cells. Each entry shows the number of reads in a peak (column) per cell (row), with rows colored by batch. Despite the variations in sequencing depth (see part **a**), Spearman based hierarchical clustering on landmark peaks does not reveal batch specific patterns.

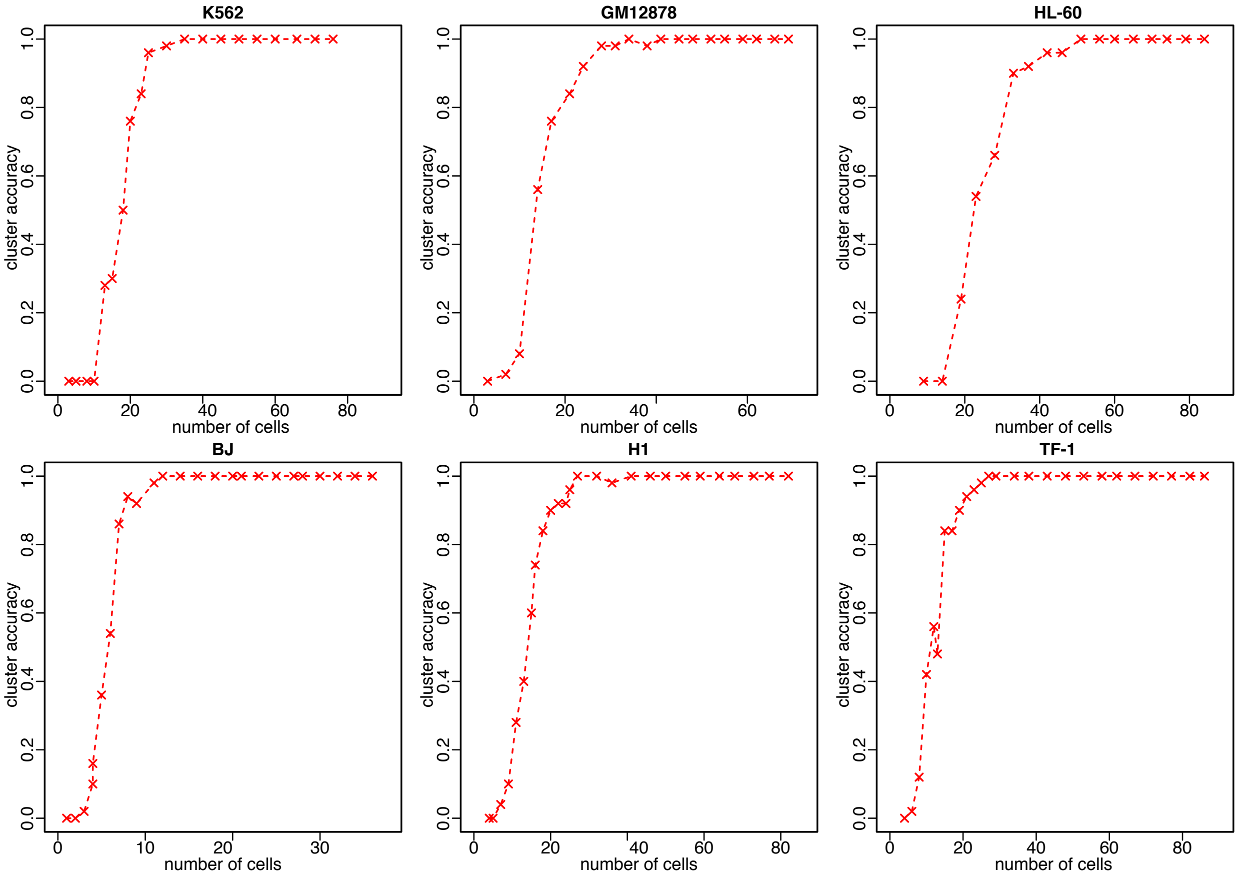

Supplementary Figure 5: ***scABC* sensitivity for detection of small sub-populations.** Each sub-population (a cell line from the *in silico* mixture) was separately downsampled, with cells removed at random, and then clustered using *scABC*. We calculated the cluster accuracy as the probability of downsampled cell sub-population forming a distinct cluster. A cluster is distinct provided that (i) at least 85% of the downsampled cell sub-population are clustered together, and (ii) within this cluster, at least 85% of the cells are from the downsampled cell sub-population. Cluster accuracy is then defined as (number of runs that results in a distinct cluster)/(total number of runs), where the total number of runs was set to 50. We observe that *scABC* identifies sub-populations that are as low as 1% of the total population. We also note that the performance is cell line specific, as we would expect dissimilar populations to be more easily recognized while similar populations are difficult to distinguish without sufficient representation.

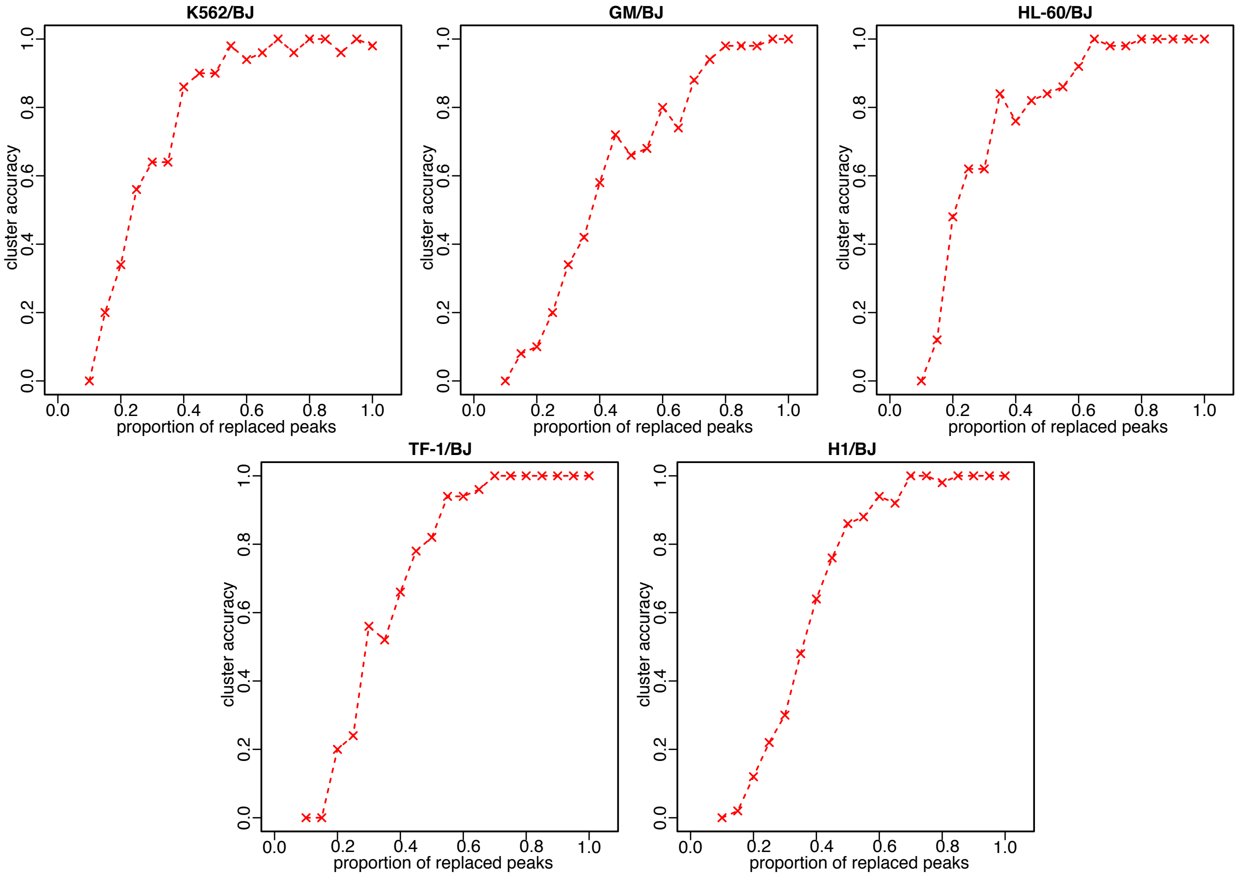

Supplementary Figure 6: ***scABC* sensitivity for detection of similar sub-populations.** We randomly picked 100 cells from each cell line of the *in silico* mixture, where sampling with replacement was performed for HL-60, TF-1, and H1 since the total number of available cells was less than 100. We then randomly divided the 100 cells into two groups of 50 cells and considered an arbitrary subset of peaks. For each cell in one group, we used a BJ cell with comparable sample depth (h_i) and replaced their read counts within the selected peaks. Cells from the two groups were then clustered using *scABC* and the cluster accuracy for cells with no replaced peak was defined similar to Supplementary Figure 5. Overall, *scABC* achieves 0.7 cluster accuracy when 30-50 percent of peaks are replaced (50%-70% cell line similarity). We emphasize that the six cell lines have a large number of peaks in common (see Fig. 1**c**) and thus, a small portion of replaced peaks derives dissimilarity between the two groups. This suggests that our analysis underestimates the true capability of *scABC* in distinguishing similar sub-populations. To better demonstrate our method performance, we also evaluated *scABC* clustering results on the RA-treated mESCs, a true biological mixture with highly similar cells compared to the *in silico* mixture (see Results).

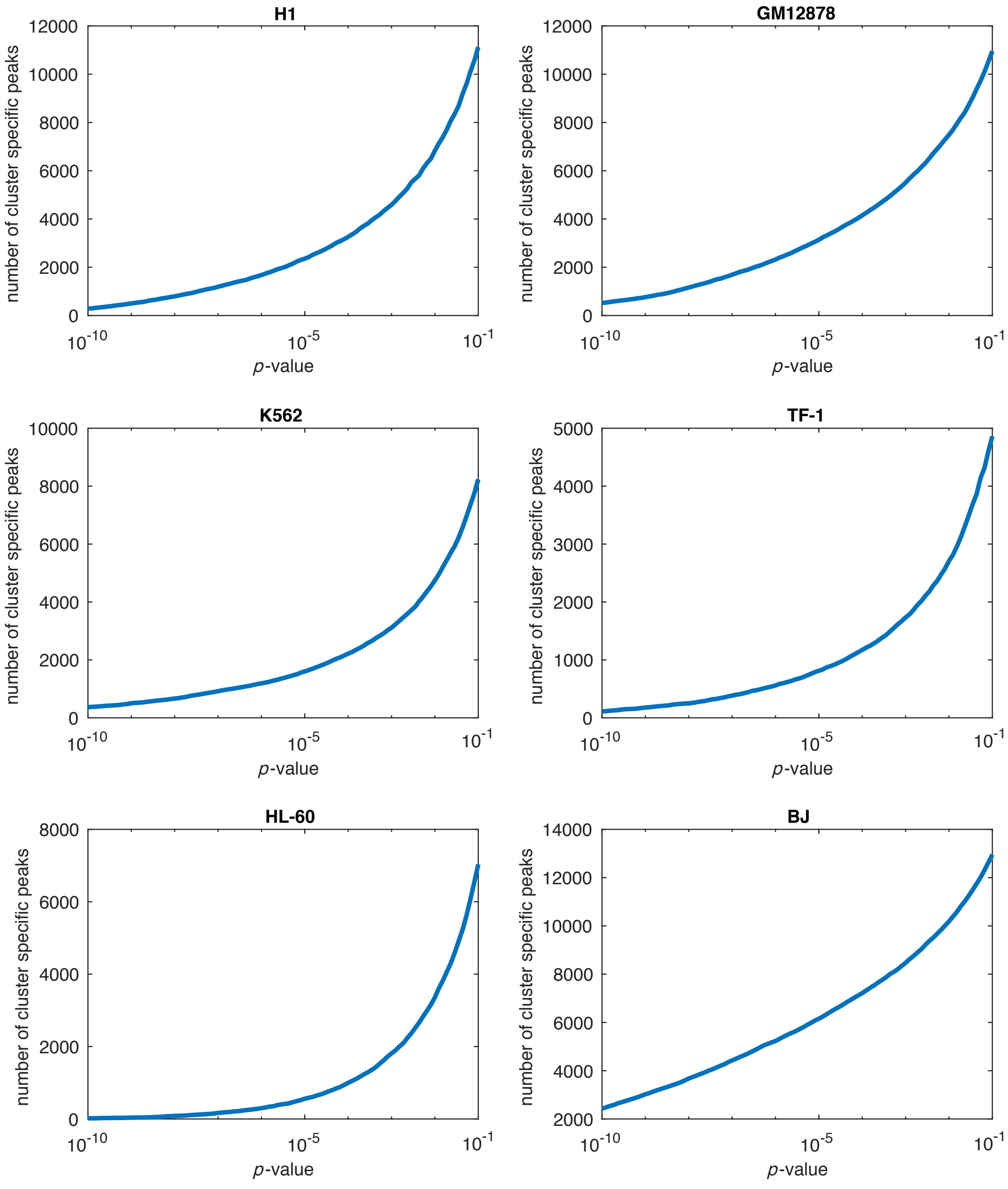

Supplementary Figure 7: **Number of *scABC*'s cluster specific peaks for the *in silico* mixture.** Here, we show the number of cluster specific peaks as a function of *p*-value cutoffs for each cell line.

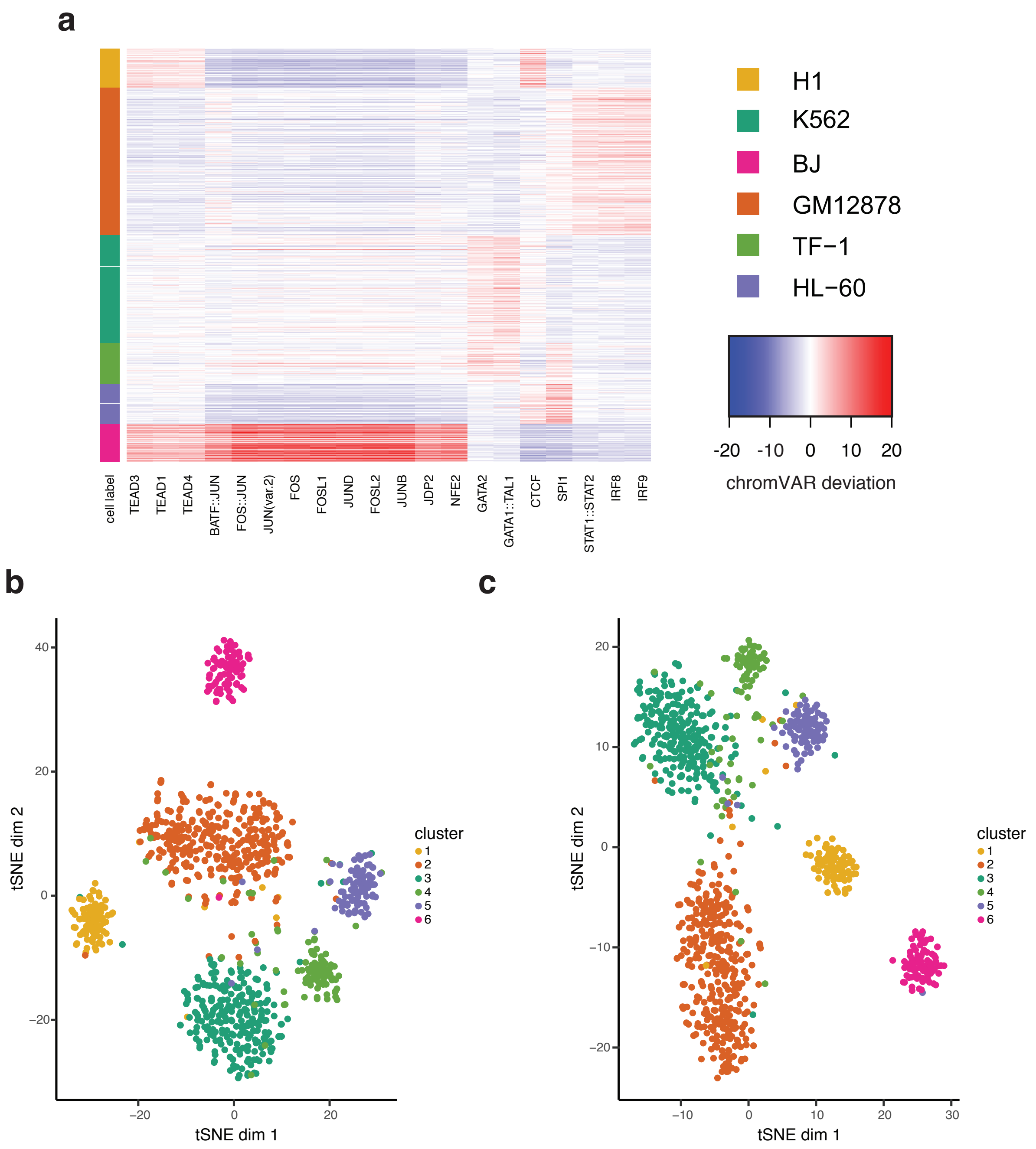

Supplementary Figure 8: **chromVAR results based on cluster specific peaks and all narrow peaks.** (**a**) chromVAR deviations for the 20 most variable TF motifs (columns), calculated using all narrow peaks from the *in silico* mixture of six cell lines (rows), with cell line assignments shown on the left. Notably, the well known H1 specific TF, POU5F1 (or other POU factors with similar motifs), is not detected while cluster specific narrow peaks capture the activity of POU motifs (Fig. 2**a**). (**b**) chromVAR *t*-SNE plot for the six clusters obtained by *scABC*, calculated using cluster specific narrow peaks, with cluster assignments shown on the right. Clear separation of the clusters indicates that the cluster specific peaks are responsible for the intrinsic variation in transcription factor motifs while using only 15% of all narrow peaks. (**c**) chromVAR *t*-SNE plot, calculated using all narrow peaks, with cluster assignments shown on the right. Here, separation of clusters is also comparable to part **b**.

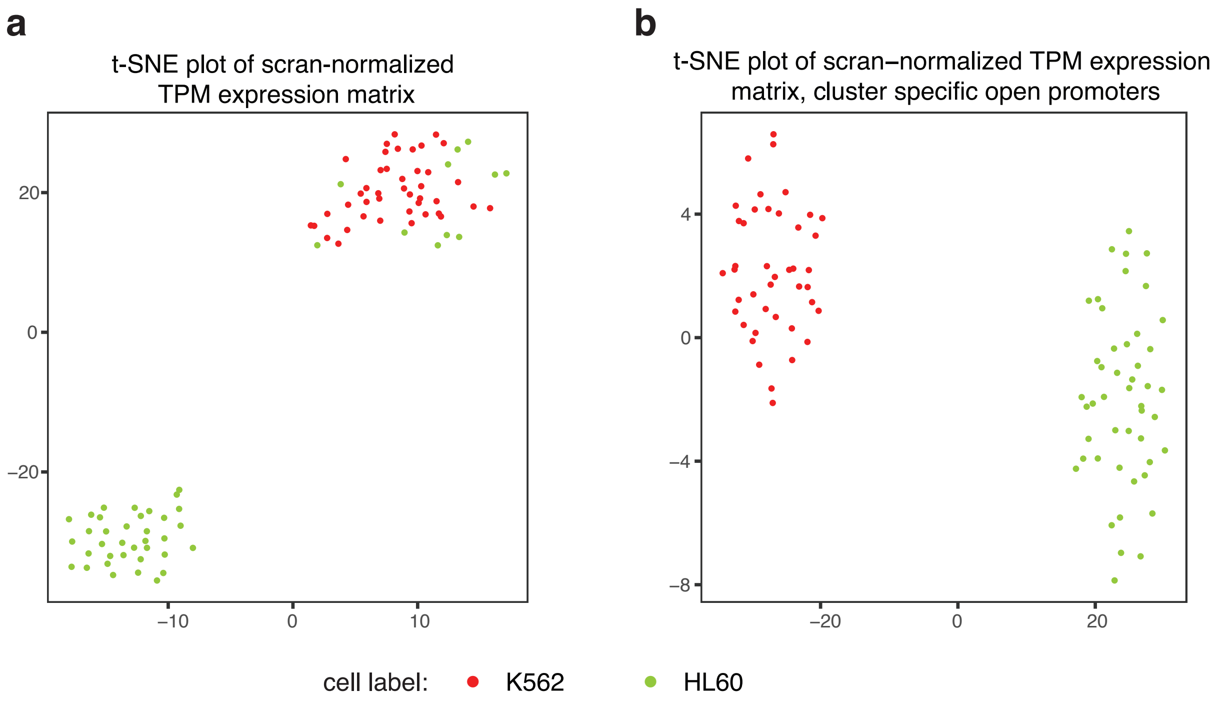

Supplementary Figure 9: ***scABC* cluster specific promoters better distinguish cells in t-SNE space.** (**a**) t-SNE plots for all genes and (**b**) genes with cluster specific open promoters. In both plots the perplexity was set to 10.

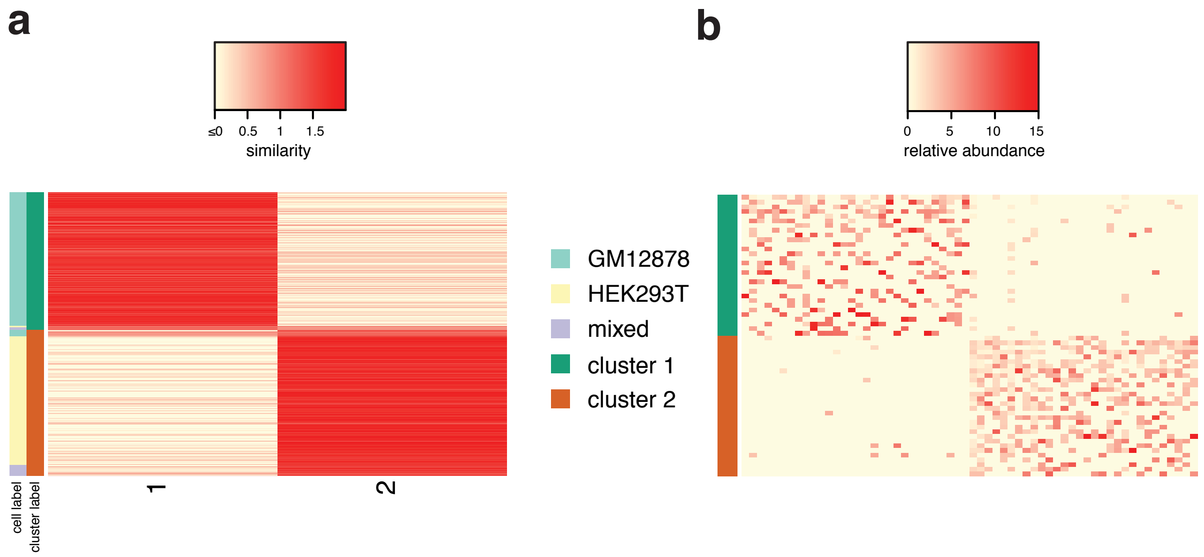

Supplementary Figure 10: ***scABC* correctly distinguishes experimentally mixed GM12878 from HEK293T cells.** *scABC* classifies GM12878/HEK293T mixture into two clusters (Supplementary Fig. 3), with one cluster dominated by GM12878 cells and the other by HEK293T. (**a**) Similarity between *scABC* calculated landmarks (columns) and cells (rows), with cluster and cell line assignments shown on the left. Cells are labeled as GM12878, HEK293T, and mixed (not determined) based on previous analyses that required known DNase I hypersensitive sites specific to each cell line [1]. (**b**) Heatmap for the relative abundance across cluster specific peaks (columns) and cells (rows), with cluster assignments shown on the left. We obtain the abundance of peaks by normalizing their read counts with respect to the sample depth h_i in each cell (see Supplementary Notes and Method). We then divide every peak abundance by the mean abundance among all cells, named relative abundance throughout the text. To simplify the presentation, for each cluster, we only depict the top 30 cluster specific peaks (i.e. the smallest p-values) and the 30 deepest samples (i.e. the largest h_i).

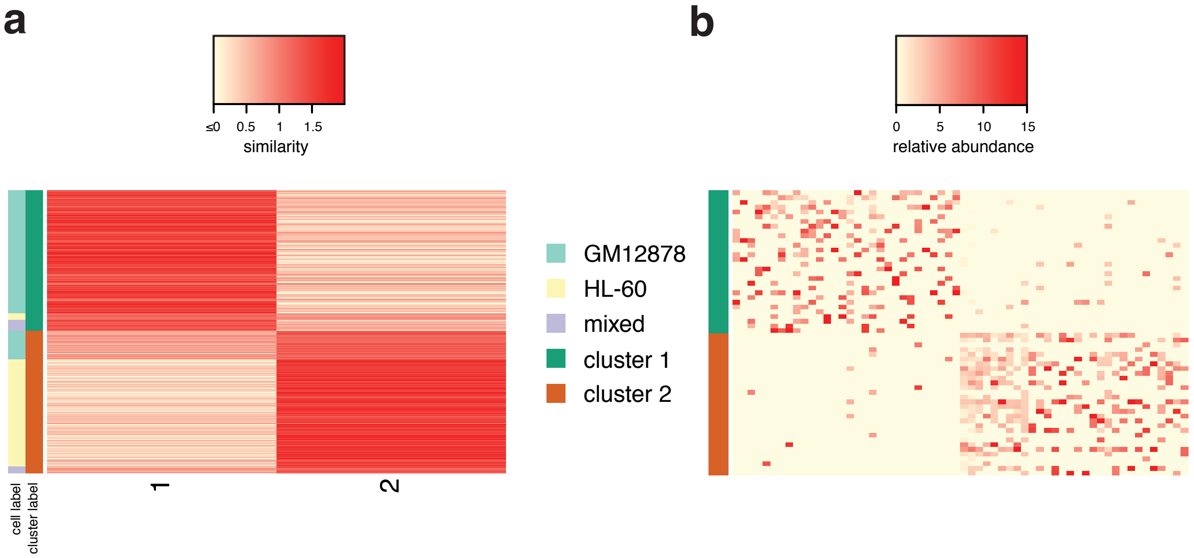

Supplementary Figure 11: ***scABC* correctly distinguishes experimentally mixed GM12878 from HL-60 cells.** *scABC* classifies GM12878/HL-60 mixture into two clusters (Supplementary Fig. 3), with one cluster dominated by GM12878 cells and the other by HL-60. (**a**) Similarity between *scABC* calculated landmarks and cells, with the similar labeling as Supplementary Figure 10. (**b**) Heatmap for the relative abundance across cluster specific peaks (columns) and cells (rows), with cluster assignments shown on the left. The top 30 specific peaks and the 30 deepest samples are depicted for each cluster.

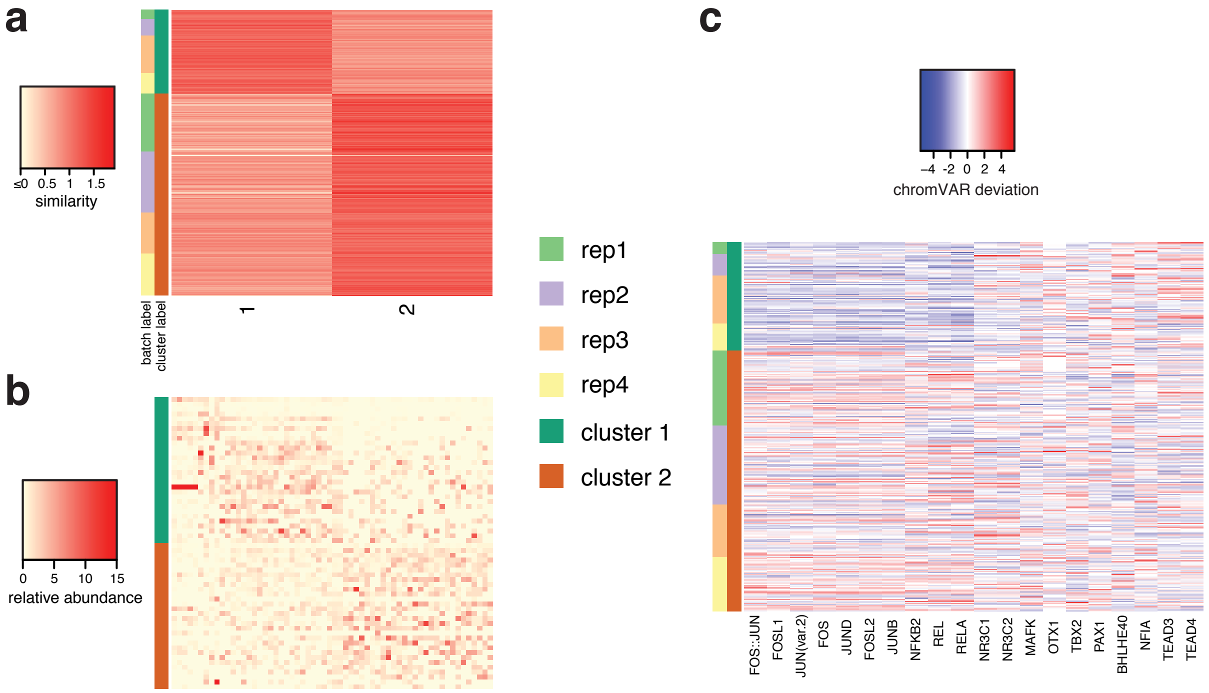

Supplementary Figure 12: ***scABC* indicates heterogeneity in GM12878 cell line.** We set the number of clusters equal to 2 and apply *scABC* to GM12878 cells for the analysis of variations observed in Supplementary Figure 4. (**a**) Similarity between cells (rows) and the two landmarks defined by *scABC* (columns), with cluster and batch assignments depicted on the left, shows the two clusters are somewhat separated. (**b**) Heatmap for relative abundance across cluster specific peaks (columns) and cells (rows), with cluster assignments shown on the left. The top 30 specific peaks and the 30 deepest samples are presented for each cluster. (**c**) chromVAR deviations for the top 20 most variable TF motifs (columns) and cells (rows), calculated using cluster specific peaks. A subset of motifs are most active in cluster 2, for instance transcription factors REL, RELA, and NFKB2 (members of the NF-κB complex) and AP-1 family. Such separation of clusters can be an indication of transcription factor heterogeneity in GM12878 cells, consistent with previous findings [2, 3].

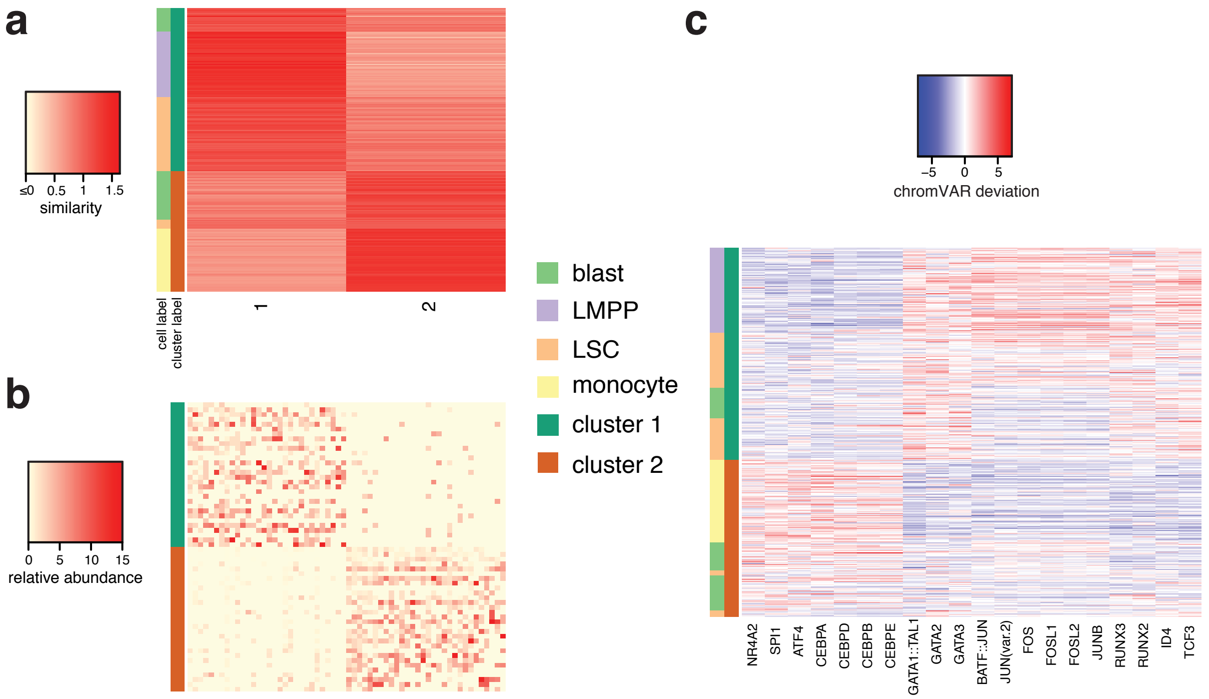

Supplementary Figure 13: ***scABC* classifies leukemic cells according to the myeloid progression**. *scABC* classifies the mixture of LSC, blast, LMPP, and monocyte cells into two clusters (Supplementary Fig. 3). One cluster corresponds to early myeloid progression and is dominated by LMPPs and the other corresponds to later myeloid progression and is dominated by monocytes. (**a**) Similarity between cells (rows) and the two landmarks defined by *scABC* (columns), with cluster and cell type assignments depicted on the left, shows clear separation between the two clusters. (**b**) Heatmap for relative abundance across cluster specific peaks (columns) and cells (rows), with cluster assignments shown on the left. The top 30 specific peaks and the 30 deepest samples are depicted for each cluster. (**c**) chromVAR deviations for the 20 most variable TF motifs (columns) and cells (rows), calculated using cluster specific peaks, identifies transcription factors specific to clusters and progression. Because we observe notable transcription factor heterogeneity in cluster 1 (AP-1 family), we increased the number of clusters for further investigation (see Supplementary Fig. 14 for detailed analysis).

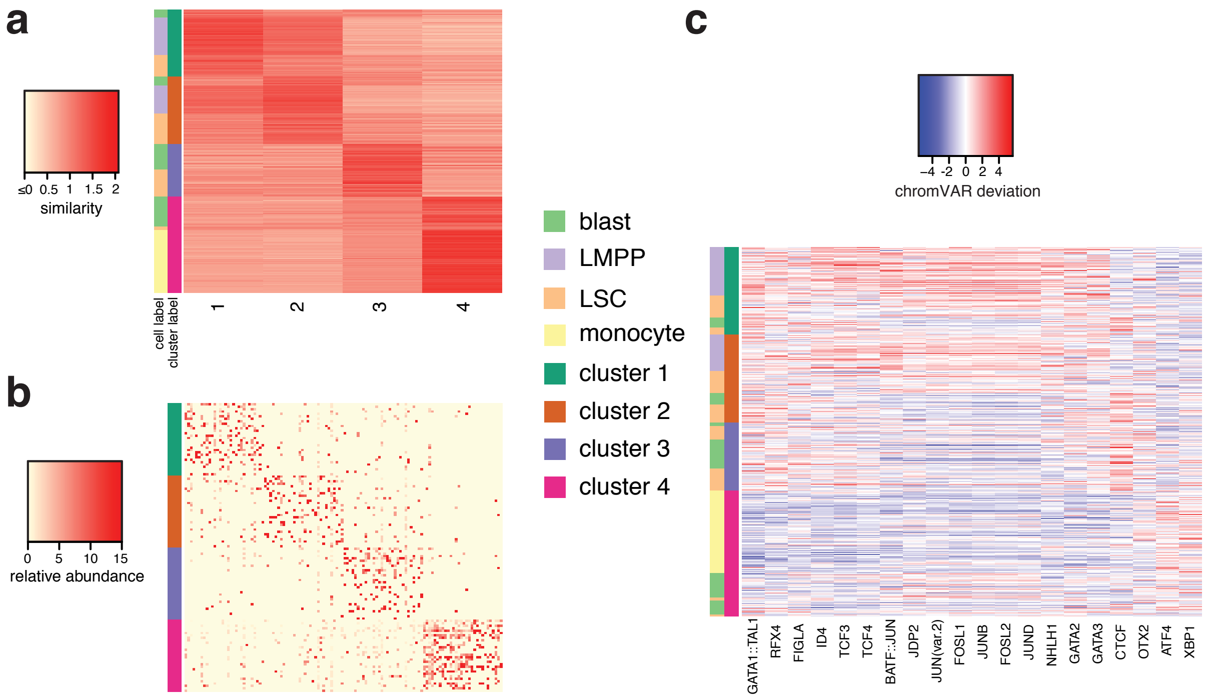

Supplementary Figure 14: **Increasing the number of clusters on the leukemic cell mixture leads to uncertainty.** (**a**) Similarity between cells (rows) and the four landmarks defined by *scABC* (columns), with cluster and cell type assignments depicted on the left, shows that clusters 1 and 2 are analogous and together represent LMPPs. The other two clusters are slightly similar, but cluster 3 is dominated by leukemia cells and cluster 4 by monocytes. (**b**) Heatmap for relative abundance across cluster specific peaks (columns) and cells (rows), with cluster assignments shown on the left. The top 30 specific peaks and the 30 deepest samples are presented for each cluster. The cluster specific peaks for clusters 1, 2, and 3 are sparse, indicating that a few cells influence these clusters. (**c**) chromVAR deviations for the top 20 TF motifs (columns) and cells (rows), calculated using cluster specific peaks, shows that cluster 4 is enriched for cluster specific TF motifs (ATF4 and XBP1). Similar motifs are enriched in clusters 1 and 2, but, this was expected since they both have analogous landmarks. Dysregulation of JUN and JUNB were previously shown to be essential for leukemic stem cell function [4], which may explain the low activity of AP-1 in cluster 3.

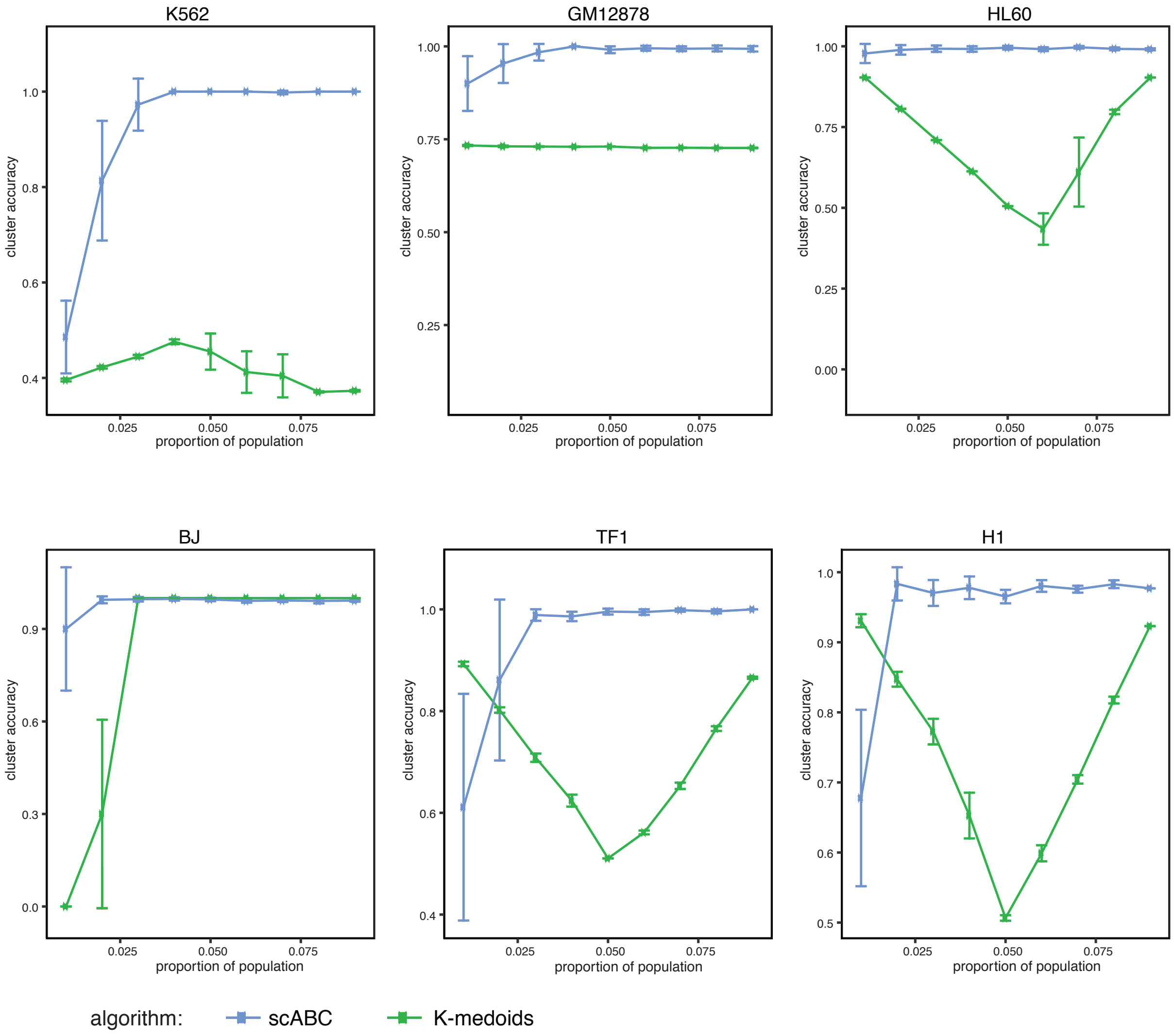

Supplementary Figure 15: **Comparison of *scABC* to naive K-medoids for the detection of small sub-populations.** For each *in silico* cell line, we took 10 random subsamples of size approximately equal to $1\%, 2\%, \ldots, 9\%$ of the total population. We clustered the cells using *scABC* and K-medoids with Spearman dissimilarity. The cluster accuracy is defined as the proportion of cells in each cell line that are assigned to a cluster that is dominated by that cell line. The points are the average cluster accuracy and the error bars defined as two times the standard error of the mean.

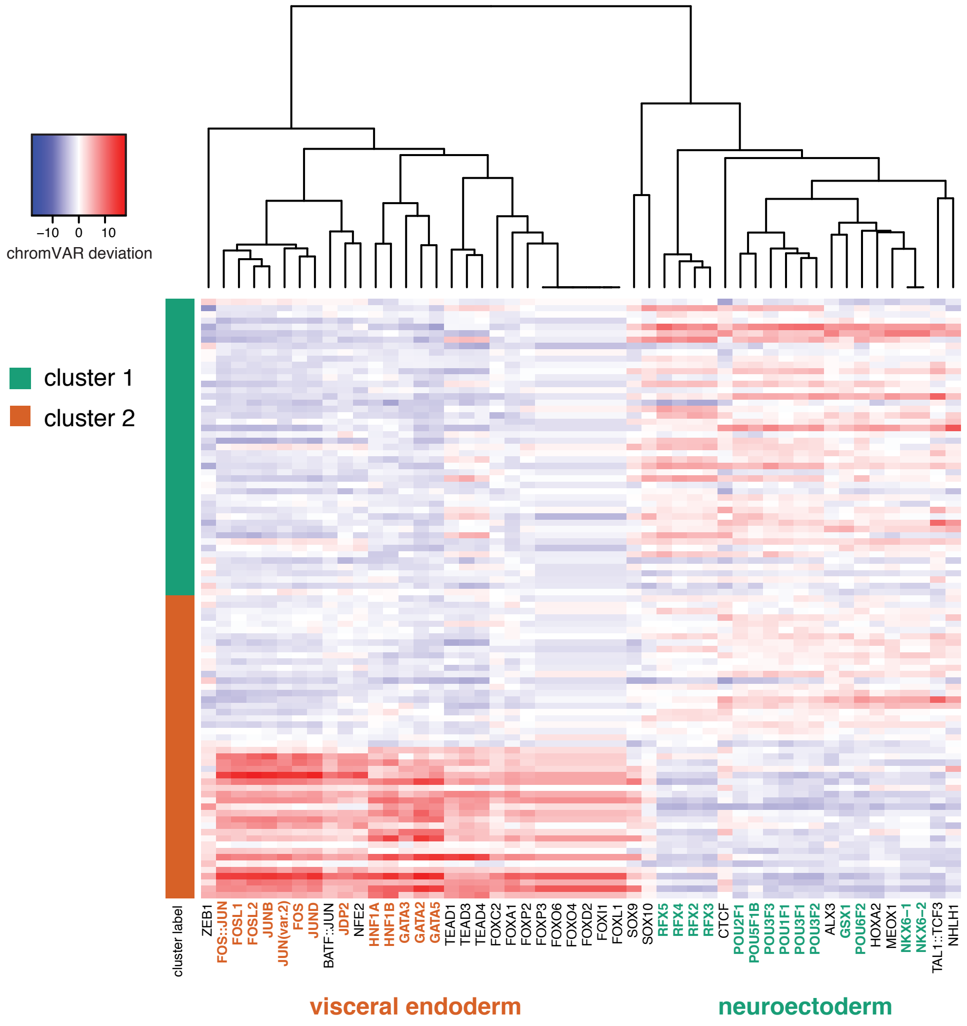

Supplementary Figure 16: **Comparison of *scABC* to naive K-medoids for the detection of similar sub-populations.** We clustered the RA-treated mESCs using K-medoids with Spearman dissimilarity. Here, we show chromVAR deviations for the 50 most variable TF motifs, calculated using all narrow peaks, with K-medoids cluster assignments depicted on the left. TFs are colored based on previous studies (Results, see [5] for RFX3). Motifs enriched in cluster 1 are partially enriched in cluster 2, suggesting that the clustering given by naive K-medoids is not biologically meaningful.

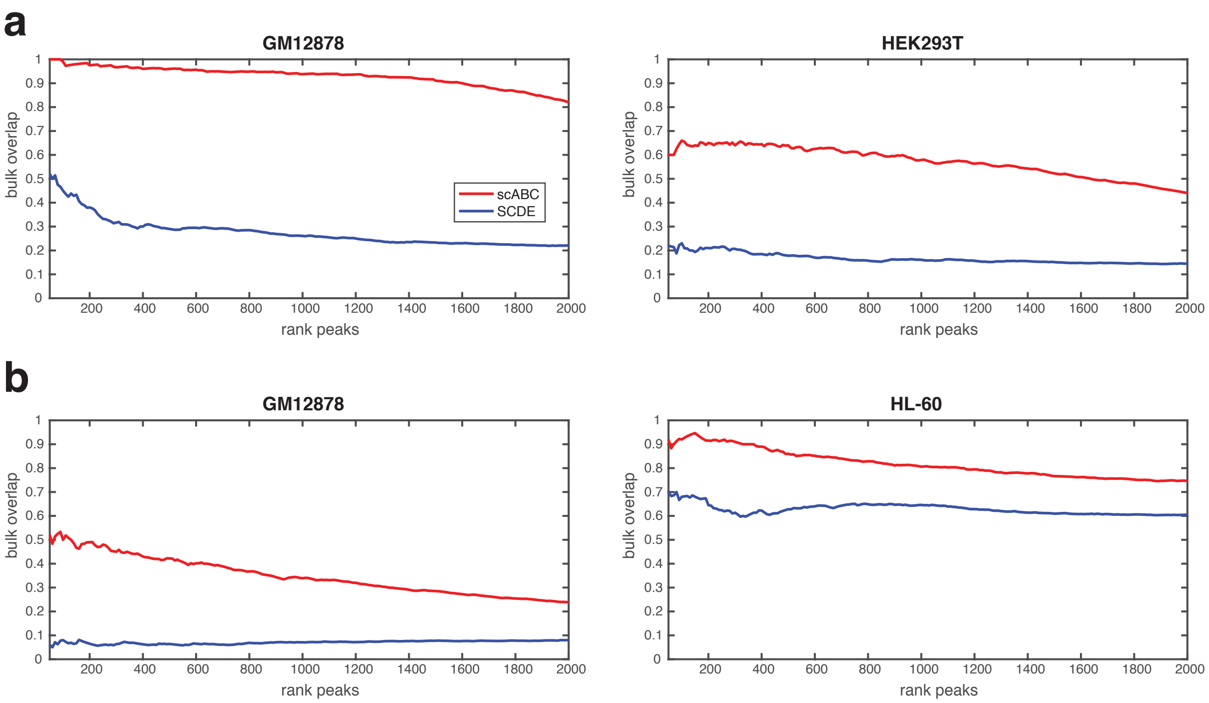

Supplementary Figure 17: **Comparison of *scABC* to SCDE for the detection of cluster specific peaks.** We applied *scABC* and SCDE to the mixtures of GM12878/HEK293T and GM12878/HL-60 and compared their cell line specific peaks with bulk data (see Supplementary Notes for detailed analysis). Note that *scABC* clustering results were used to identify the cell lines. For each mixture, we calculated the proportion of cell line specific peaks that are differentially accessible in the respective bulk measurements, named bulk overlap. (**a**) *scABC* (red) and SCDE (blue) bulk overlaps for each cell line in GM12878/HEK293T mixture. Peaks are ranked based on *scABC* p-value and SCDE z-score. (**b**) *scABC* and SCDE bulk overlaps for each cell line in GM12878/HL-60 mixture. In all cases, *scABC* demonstrates higher overlap with cell type specific peaks from the respective bulk data.

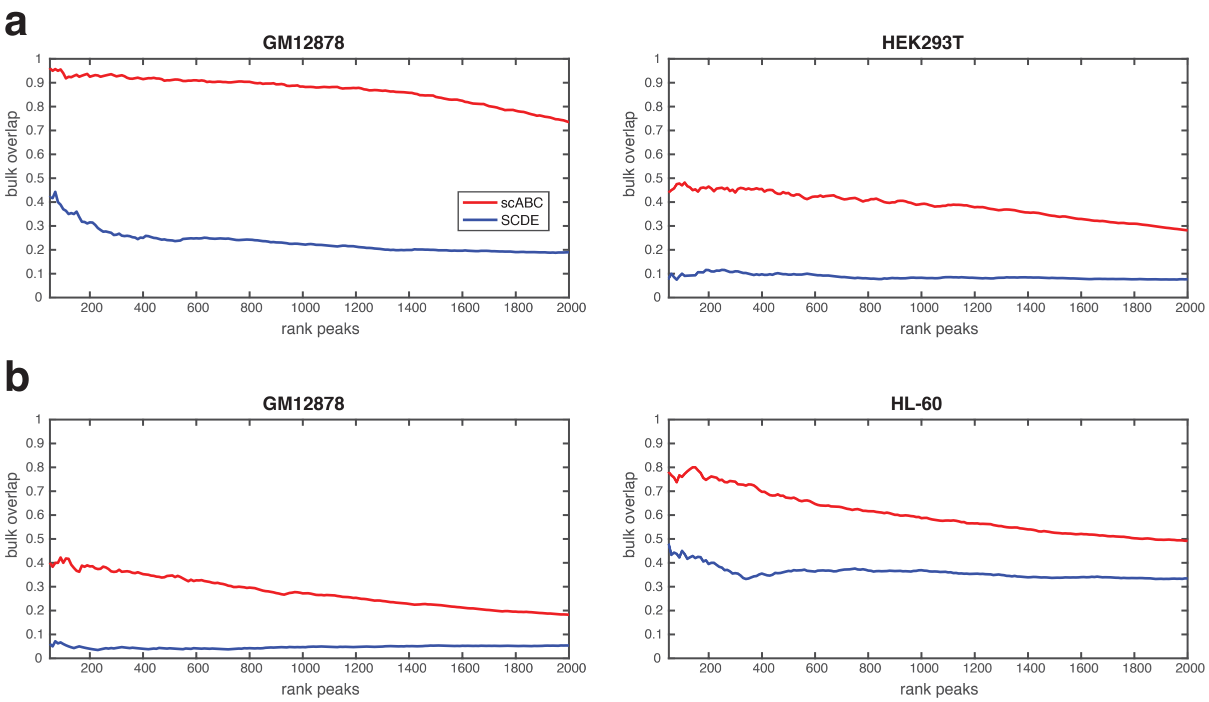

Supplementary Figure 18: **Imposing stricter condition for the determination of bulk differentially open peaks leads to similar results.** We performed the same analysis as Supplementary Figure 17, but, changed the threshold T from 2 to 3 (Supplementary Notes). (**a**) *scABC* (red) and SCDE (blue) bulk overlaps for each cell line in GM12878/HEK293T mixture. (**b**) *scABC* and SCDE bulk overlaps for each cell line in GM12878/HL-60 mixture. Although increasing T results in less overall overlap for both methods, *scABC* still achieves higher overlap than SCDE.

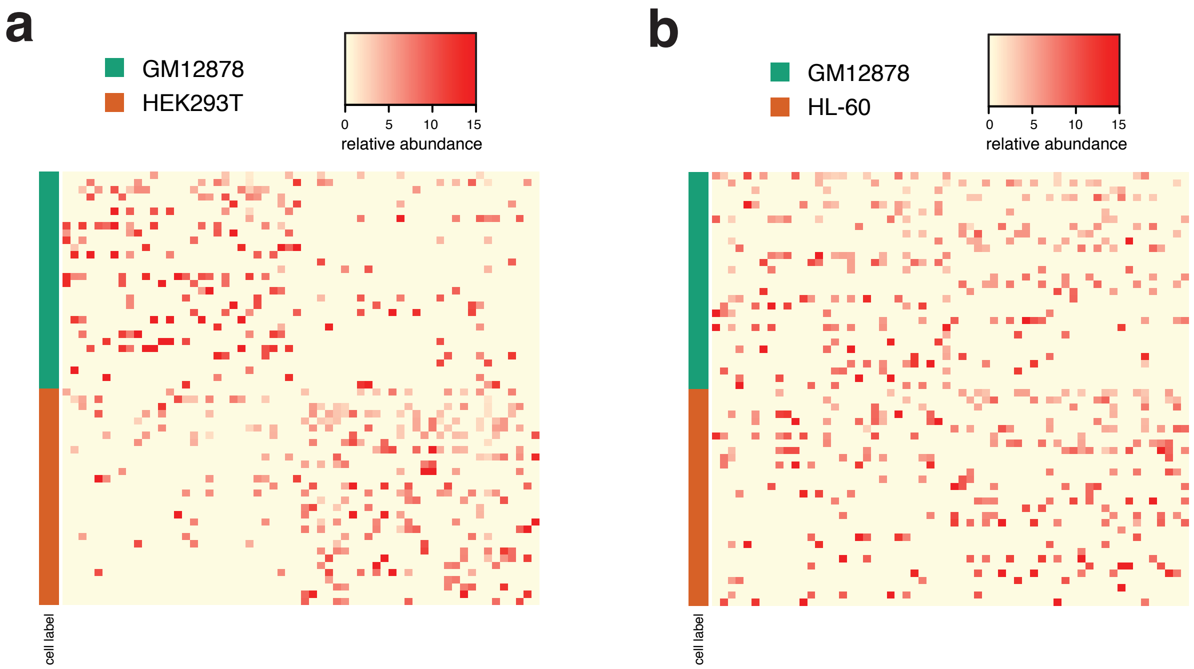

Supplementary Figure 19: **SCDE's cluster specific peaks are not well distinguished.** We used SCDE to obtain peaks specific to each cell line (identified by *scABC*) in GM12878/HEK293T and GM12878/HL-60 mixtures. Here, we present the heatmap for the relative abundance across cell line specific peaks (columns) and cells (rows), with cell line assignments shown on the left. The top 30 specific peaks and the 30 deepest samples are depicted for each cell line. Part **a** shows the heatmap for GM12878/HEK293T mixture and part **b** for GM12878/HL-60.

2 Supplementary Tables

DNase-seq \ peaks	top 50,000	condition 1	condition 2
ENCSR000EKN	28%	86%	87%
ENCSR000EKO	30%	97%	97%
ENCSR000EKP	31%	95%	96%
ENCSR000EKQ	32%	96%	96%
ENCSR000EKR	28%	95%	96%
ENCSR000EKS	30%	97%	97%
ENCSR000EPC	20%	93%	94%

Supplementary Table 1: Genome-wide accessibility comparison of scATAC-seq and DNase-seq in K562 cells. The first column shows ENCODE accession number for DNase-seq experiments and column two represents the percentage of top 50,000 peaks, obtained from aggregated scATAC-seq data, that have overlaps ($\geq 10\%$) with DNase-seq open regions. Condition 1 refers to peaks containing 1 or more reads in at least 10 cells and condition 2 requires minimum 2 reads in at least 10 cells. Total number of peaks selected by conditions 1 and 2 are 19,331 and 17,484. Columns 3 and 4 provide the fraction of these peaks overlapping with the bulk data.

cluster \ cell line	K562	GM12878	HL-60	BJ	TF-1	H1
cluster 1	252	0	0	0	0	2
cluster 2	0	344	1	0	0	0
cluster 3	0	0	92	0	0	0
cluster 4	0	0	0	89	0	0
cluster 5	0	0	0	0	96	0
cluster 6	0	1	0	0	0	89

Supplementary Table 2: *scABC* classifies the *in silico* mixture of six cell lines into six clusters. Entries provide the number of cells of each cell line (columns) for clusters (rows).

cluster \ batch	rep1	rep2	rep3	rep4
cluster 1	1	0	0	0
cluster 2	12	23	46	28
cluster 3	68	71	46	48
cluster 4	0	0	2	0

Supplementary Table 3: The number of cells for each of four clusters defined by *scABC* and four batches for GM12878 cells. The majority of cells falls within clusters 2 and 3 that are not specific to any replicates, indicating the robustness of *scABC* to bach effects.

batch / cluster	rep1	rep2	rep3	rep4
cluster 1	1	0	0	0
cluster 2	1	0	0	0
cluster 3	1	0	0	0
cluster 4	1	0	0	0
cluster 5	1	0	0	0
cluster 6	9	23	43	30
cluster 7	67	71	45	46
cluster 8	0	0	3	0
cluster 9	0	0	1	0
cluster 10	0	0	2	0

Supplementary Table 4: The number of cells for each of ten clusters defined by *scABC* and four batches for GM12878 cells. We do not observe any clear batch effects while imposing clusters to be greater than batches in number.

cell type / cluster	LMPP	monocyte	SU353-LSC	SU353-blast	SU070-LSC	SU070-blast
cluster 1	90	0	44	32	58	0
cluster 2	0	87	7	37	5	30

Supplementary Table 5: The number of cells corresponding to each of the two clusters defined by *scABC* for the mixture of leukemic cells. Columns show the cell type and SU353 and SU070 are the patients IDs of the donors suffering from acute myeloid leukemia.

cell type / cluster	LMPP	monocyte	SU353-LSC	SU353-blast	SU070-LSC	SU070-blast
cluster 1	51	0	7	11	23	0
cluster 2	39	0	19	12	23	0
cluster 3	0	0	23	31	14	4
cluster 4	0	87	2	15	3	26

Supplementary Table 6: The number of cells corresponding to each of the four clusters defined by *scABC* for the mixture of leukemic cells.

cell type / cluster	K562	GM12878	HL-60	BJ	TF-1	H1
cluster 1	0	0	0	0	0	88
cluster 2	251	0	2	0	1	1
cluster 3	0	344	0	0	2	2
cluster 4	0	1	0	0	93	0
cluster 5	1	0	91	0	0	0
cluster 6	0	0	0	89	0	0

Supplementary Table 7: The number of cells from the *in silico* mixture designated to the six clusters defined by naive K-medoids with Spearman dissimilarity measure. K-medoids arrives at 10 misclassifications, slightly worse than *scABC* (Supplementary Table 2).

cell line / cluster	K562	GM12878	HL-60	BJ	TF-1	H1
cluster 1	7	60	93	0	4	17
cluster 2	0	0	0	0	92	0
cluster 3	0	5	0	0	0	0
cluster 4	0	280	0	0	0	74
cluster 5	245	0	0	0	0	0
cluster 6	0	0	0	89	0	0

Supplementary Table 8: The number of cells from the *in silico* mixture designated to the six clusters defined by K-means on the $\log$ TPM matrix. K-means results in 167 misclassifications and cannot separate GM12878 from HL-60 and also GM12878 from H1.

cell type / cluster	K562	GM12878	HL-60	BJ	TF-1	H1
cluster 1	0	5	2	0	0	85
cluster 2	251	3	14	0	0	2
cluster 3	0	337	2	0	0	1
cluster 4	1	0	74	0	0	2
cluster 5	0	0	0	89	0	1
cluster 6	0	0	1	0	96	0

Supplementary Table 9: We binned the reads into large genomic intervals (100 kb) based on the windowCounts function in the csaw bioconductor package [6] and removed regions that had no counts across cells. We then used these large intervals to cluster cells using naive K-medoids with Spearman dissimilarity measure. Here, we show the number of cells from the six cell line mixture corresponding to the six clusters defined by K-medoids. In total 34 cells are misclassified, higher than the 10 misclassifications by naive K-medoids on the unbinned counts (Supplementary Table 7).

cell type / cluster	K562	GM12878	HL-60	BJ	TF-1	H1
cluster 1	67	111	6	16	29	21
cluster 2	18	50	38	0	46	42
cluster 3	0	0	0	70	0	0
cluster 4	0	172	0	0	0	0
cluster 5	1	10	22	0	7	6
cluster 6	166	2	27	3	14	22

Supplementary Table 10: Similar to the previous table, we used binned counts for the *in silico* mixture and clustered cells using K-means on the $\log$ TPM matrix. Except for BJ, other cell lines cannot be distinguished.

cell line / cluster	K562	GM12878	HL-60	BJ	TF-1	H1
cluster 1	0	345	2	14	0	8
cluster 2	5	0	7	1	2	9
cluster 3	247	0	0	0	0	0
cluster 4	0	0	0	0	0	74
cluster 5	0	0	0	0	94	0
cluster 6	0	0	84	74	0	0

Supplementary Table 11: The number of cells from the *in silico* mixture designated to the six clusters defined by SC3. SC3 leads to 122 misclassifications and cannot separate HL-60 from BJ, two distinct cell types.

cell line / cluster	K562	GM12878	HL-60	BJ	TF-1	H1
cluster 1	16	145	11	80	18	27
cluster 2	171	0	57	0	18	0
cluster 3	63	1	25	9	60	0
cluster 4	0	108	0	0	0	38
cluster 5	0	80	0	0	0	19
cluster 6	2	11	0	0	0	7

Supplementary Table 12: The number of cells from the *in silico* mixture designated to the six clusters defined by the cluster_infomap function in the igraph R package. All cell lines are mixed and cannot be separated.

algorithm / cluster	*scABC*	naive K-medoids
cluster 1	67	47
cluster 2	28	48

Supplementary Table 13: Clustering results for RA-treated mESCs obtained from *scABC* and naive K-medoids with Spearman dissimilarity with $K = 2$ for both methods. K-medoids divides the mixture into two groups with the similar number of cells. These two groups are not in agreement with the analysis of TF enrichment (Supplementary Fig. 16).

λ \ c	1	2	3	4	5	6	7	8	9	10	11	12	13	14	15	20	25	30	40	50
0.001	962	962	962	962	962	962	962	962	962	962	962	962	962	962	962	962	962	871	871	514
0.01	962	962	962	962	962	962	962	962	962	962	962	962	962	962	962	962	962	871	871	514
0.02	962	962	962	962	962	962	962	962	962	962	962	962	962	962	962	962	962	871	871	514
0.05	962	962	962	962	962	962	962	962	962	962	962	962	962	962	962	962	962	871	871	515
0.1	962	962	962	962	962	962	962	962	962	962	962	962	962	962	962	962	962	871	871	513
0.2	962	962	962	962	962	962	962	962	962	962	962	962	962	962	962	962	962	962	871	455
0.5	962	962	962	962	962	962	962	962	962	962	962	962	962	962	962	962	962	962	962	962
1	962	962	962	962	962	962	962	962	962	962	962	962	962	962	962	962	962	962	962	962
10	962	962	962	962	962	962	962	962	962	962	962	962	962	962	962	962	962	962	962	962

Supplementary Table 14: *scABC*, applied to the *in silico* mixture of six cell lines, is robust to ranges of various λ and c in the weighting scheme. Rows and columns correspond to λ and c, respectively. Entries represent the number of cells that are correctly clustered (out of the 966 total cells), assuming the majority of cluster's cells with the same type shows the cluster identity.

3 Supplementary Note

3.1 Data processing

Single cell chromatin accessibility data

The scATAC-seq data from [2] was used to construct a *in silico* heterogeneous population, publicly available in the Gene Expression Omnibus (GEO) under the accession number GSE65360. Single-cell data for LSC, blast, LMPP, and monocyte cells are available under GEO accession GSE74310 [7]. GSE68103 also provides sequencing data for the mixtures of GM12878/HEK293T and GM12878/HL-60 cell lines [1]. In addition, we generated 96 RA-treated mESCs (see Results and Methods), available at GEO with the accession GSE107651.

Read alignment and peak calling

We used the Kundaje pipeline (`https://github.com/kundajelab/atac_dnase_pipelines`) to process the raw scATAC-seq reads, aligning reads to hg19 and removing duplicates (mm9 for RA-treated mESCs). Alternative schemes may improve data preprocessing, however, our main objective is to determine cell identities using only the aligned sequencing reads as inputs. Similar to previous studies, such as [2], we employed MACS2 to perform peak calling on accessibility data aggregated across all cells [8]. MACS2 generates i) narrow peaks generally called for transcription factor binding sites and ii) broad peaks suitable for detection of functional regulatory elements such as promoters and enhancers. We utilized both narrow and broad regions, called gapped peaks, to better classify cell identities.

For the GM12878/HEK293T and GM12878/HL-60 mixtures, sequenced through combinatorial cellular indexing, we used the processed data available through GEO, accession number GSE68103.

Reliable peaks

Given the sparse nature of single-cell data, we suspected that open chromatin peaks, identified using aggregated data, may differ from the bulk data. To characterize the variability between bulk and merged scATAC-seq data, we focused on K562 myeloid leukemia cell line because it has numerous publicly available bulk datasets. Specifically, we compared aggregated single cell accessibility peaks to DNase hypersensitivity regions retrieved from the ENCODE project, that we refer to as bulk peaks. As indicated in Supplementary Table 1, roughly 30% of the top 50,000 peaks from aggregated data have overlaps with the bulk peaks. We assume that peaks A and B intersect if B overlaps at least 10% of A, or vice versa. Peaks that appear in multiple cells are naturally more reliable than peaks that appear in only one single cell, similar to SNP calling in single cell analysis [9]. Therefore, we focused on the subset of peaks that are present (1 or more reads) in at least 10 single cells, resulting in 19,331 peaks. The peak intersections between the selected subset and bulk data was significantly improved compared to the top 50,000 peaks of the aggregated data (columns 3 in Supplementary Table 1). Increasing the minimum number of reads to 2 slightly reduces peaks to 17,484 and slightly improves the intersections (columns 4 in Table 1). We also observed that an additional growth in the number of required reads or cells notably decreases reliable peaks.

For the analysis of *in silico* cell lines, GM12878/HEK293T, and GM12878/HL-60 mixtures, we used peaks that are presented in $\geq$ 10 cells with $\geq$ 2 reads. We obtained 94,934 peaks for the *in silico* cell lines, 12,938 for GM12878/HEK293T mixture, and 10,431 for GM12878/HL-60 mixture. However, for the leukemia mixture and RA-treated mESCs, we considered peaks with minimum 2 reads in at least 5 cells since the leukemia mixture has less than 91 extremely sparse samples for each cell type and only 95 RA-treated mESCs are available. In total, we arrived at 52,336 peaks for the leukemia mixture and 9,661 for RA-treated mESCs.

Sample quality and depth

We only considered scATAC-seq samples that have at least $\min(500,\ \text{number of reliable peaks}/50)$ read counts across reliable peaks, leading to 966 cells for the *in silico* mixture of six cell lines, 95 for the RA-treated mESCs, and 390 for the leukemia, LMPP, and monocyte mixture. However, we did not apply this thresholding to GM12878/HEK293T (748 cells) and GM12878/HL-60 (700 cells) mixtures due to the extreme sparsity of these datasets (Supplementary

Figure 1). We define background regions as 500,000 base pairs upstream and downstream from reliable peak centers. We use the median of background reads for each cell, denoted as h_i in Method, to represent sample depth.

Single cell expression data and analysis

Raw sequencing files were downloaded from SRA and mapped with STAR [10] to hg38 using the GENCODE v24 transcriptome annotation. Gene counts were obtained with HTSeq [11] using the intersection-nonempty mode and converted to transcripts per million (TPM) for subsequent analysis. We corrected for batch effects with the R package scran [12].

3.2 Choosing the number of clusters

To systematically determine the number of clusters for the weighted K-medoids algorithm, we take advantage of the gap statistic [13] with some modifications. Let W_K represent a measure for within-cluster dispersion, which is a function of the cluster number K. The gap statistic compares the curve $\log W_K$ between the data and the expectation under the null reference distribution of the data [13]. The number of clusters K can be determined using the following procedure:

Step 1: Perform the weighted K-medoids clustering algorithm on the observed data, varying the total number of clusters from 1 to the maximum number of clusters N, $K = 1, \cdots, N$, and use the weighted K-medoids optimized objective function in the last iteration as W_K.

Step 2: Generate B reference data sets, by performing permutation for each feature (peak). Let Y denote the cell$\times$feature data matrix, the permutation shuffles the entries in each column. Perform the weighted K-medoids clustering algorithm on the B reference data sets, varying the total number of clusters, and calculate the weighted K-medoids objective W^*_{Kb}, for $b = 1, \cdots, B$ and $K = 1, \cdots, N$.

Step 3: compute the estimated gap statistics

$$\text{Gap}(K) = \frac{1}{B}\sum_b \log W^*_{Kb} - \log W_K,$$

and the standard deviation

$$\text{sd(K)} = \sqrt{\frac{1}{B}\sum_b (\log W^*_{Kb} - \bar{l})^2},$$

where $\bar{l} = \sum_b \log W^*_{Kb}/B$. Finally we choose the number of clusters by

$$\hat{K} = \text{smallest } K \text{ such that } \text{Gap}(K) \geq \text{Gap}(K+1) - \text{sd}(K+1)$$

We made two major modifications compared with the originally proposed gap statistic [13]. First, we use the objective function of the weighted K-medoids algorithm as the within cluster dispersion W_K. The original version uses the within cluster sum of pairwise distance. This modification accounts for the presence of noisy samples with low sequencing depth. Second, we perform permutation to generate the null reference distribution. The originally version uses simulated data uniformly distributed over a rectangle containing the data. This modification accounts for the sparsity in single cell data.

In practice, we found that if the data matrix is excessively sparse, even performing permutation may not work well. Therefore we only use the top $5,000$ peaks, those with the highest read counts when summing reads across all cells, to select the number of clusters.

3.3 Computation of the maximum likelihood estimate of regression coefficient

The maximum likelihood estimate $\boldsymbol{\beta}^{mle}$ can be computed using the Fisher scoring method [14]. Let X denote the $n \times K$ design matrix, where n is the number of cells and K is the number of clusters, with entry $x_{ik} = 1$ if cell i

belongs to cluster k and $x_{ik} = 0$ otherwise. The computation can be implemented separately for each peak. Let Y_r and $\boldsymbol{\beta}_r$ represent the read counts for each peak and coefficients for cell r, respectively.

In Fisher scoring method, $\boldsymbol{\beta}_r$ is updated iteratively. At the $(t+1)$th iteration, the update for $\boldsymbol{\beta}_r$ is

$$\boldsymbol{\beta}_r^{(t+1)} = \boldsymbol{\beta}_r^{(t)} + (X^T W_r^{(t)} X)^{-1} X^T (Y_r - \boldsymbol{\mu}_r^{(t)}),$$

where $\boldsymbol{\mu}_r^{(t)}$ is a column vector with the ith entry $\mu_{ri}^{(t)} = h_i \exp(\sum_{k=1}^{K} x_{ik} \beta_{rk}^{(t)})$, for $i = 1, \cdots, n$; $W_r^{(t)}$ is a $n \times n$ diagonal matrix with entries $(\mu_{ri}^{(t)})_{i=1,\cdots,n}$ on the diagonal. We initialize the algorithm from a vector of all zeros and iterate until convergence. In the iterations, the matrix $X^T W_r^{(t)} X$ can be close to singular for some peaks, especially for the peaks with low read counts. We do not incorporate those peaks in estimating the empirical prior σ_r.

3.4 Computation of the maximum a posteriori estimate

The maximum a posteriori estimate $\hat{\boldsymbol{\beta}}$ can be computed using an iteratively reweighted ridge regression algorithm [15, 16]. With a slight abuse of notation, let X denote the expanded $n \times (K+1)$ design matrix with the first column all 1s, representing the presence of intercept. Let D represent a $(K+1) \times (K+1)$ diagonal matrix. The first diagonal entry in D is 0, as we assume that the intercept is fixed, and the other entries are $1/\sigma_k^2$, for $k = 1, \cdots, K$. The computation can be implemented separately for each peak.

At the $(t+1)$th iteration, the update for $\boldsymbol{\beta}_r$ is

$$\boldsymbol{\beta}_r^{(t+1)} = (X^T W_r^{(t)} X + D)^{-1} X^T W_r^{(t)} X \boldsymbol{\beta}_r^{(t)} + (X^T W_r^{(t)} X + D)^{-1} X^T W_r^{(t)} Z_r^{(t)},$$

where $Z_r^{(t)}$ is a column vector with the ith entry $z_{ri}^{(t)} = (y_{ri} - \mu_{ri}^{(t)})/\mu_{ri}^{(t)}$, $\mu_{ri}^{(t)} = h_i \exp(\beta_0^{(t)} + \sum_{k=1}^{K} x_{ik} \beta_{rk}^{(t)})$, and $W_r^{(t)}$ is a diagonal matrix with $(\mu_{ri}^{(t)})_{i=1,\cdots,n}$ on the diagonal.

3.5 Computation of the standard error

We use $\hat{\Sigma}_r$ as the covariance matrix for the maximum a posteriori estimate $\hat{\boldsymbol{\beta}}_r$ [15, 17]:

$$\hat{\Sigma}_r = (X^T \hat{W}_r X + D)^{-1} (X^T \hat{W}_r X)(X^T \hat{W}_r X + D)^{-1},$$

where $\hat{W}_r$ is a diagonal matrix with $(\hat{\mu}_{ri})_{i=1,\cdots,n}$ on the diagonal, and $\hat{\mu}_{ri} = h_i \exp(\hat{\beta}_0 + \sum_{k=1}^{K} x_{ik} \hat{\beta}_{rk})$. Let $c_{kk'}$ denote a column vector representing a contrast, such that $c_{kk'}^T \boldsymbol{\beta}_r = \beta_{rk} - \beta_{rk'}$. Then the standard error

$$SE(\beta_{rk} - \beta_{rk'}) = \sqrt{c_{kk'}^T \hat{\Sigma}_r c_{kk'}}$$

3.6 Experimental mixtures of single cells

Darren et al. [1] proposed a method that integrates combinatorial cellular indexing and ATAC-seq to measure chromatin accessibility in numerous single cells. They applied their method to an experimental mixture of GM12878 and HEK293T cell lines, leading to chromatin accessibility profiles of 748 cells with a very low sequencing depth (median: 1,685 unique mapped reads, see Supplementary Fig. 1). They also obtained 700 cells from an experimental mixture of GM12878 and HL-60 (median sequencing depth: 1,390 unique mapped reads). Cell identities (GM12878 against HEK293T or HL-60) are not captured through the combinatorial cellular indexing and therefore computational approaches were needed for the inference of cell lines. In particular, Darren et al. [1] used bulk DNase-seq data to obtain cell line specific DNase I hypersensitive sites (DHSs) and the cell identities were predicted based on the fraction of reads mapped to the DHSs. They also showed that cells can be correctly separated when cell line specificities of the DHSs are not given. In both cases, the cell classification requires pre-defined DHSs for each mixture, which can be hard to obtain for complex and unknown cell populations. In contrast to this method, *scABC* only utilizes single cell accessibility data to cluster cells.

To evaluate our unsupervised clustering algorithm on experimental mixtures, *scABC* was applied to the GM12878/HEK293T and GM12878/HL-60 cells, where $K = 2$ clusters were detected for both mixtures (Supplementary Fig. 3). As illustrated in Supplementary Figure 10, landmarks distinguish GM12878 from HEK293T cells with a few misclassifications compared to the cell line assignments of [1]. Note that our mispredictions could be correct since the ground truth is not known and the computationally determined cell lines in [1] may differ from the ground truth. In addition, our procedure provides cell line specific peaks, which were determined by bulk data in the previous analysis. In a similar fashion, Supplementary Figure 11 exhibits our clustering results on GM12878/HL-60 cells, which are in a good agreement with [1]. Notably, cells are well clustered considering that GM12878 and HL-60 are two analogous cell lines and this mixture has lower sequencing depth compared to GM12878/HEK293T.

3.7 The evaluation of cluster specific peaks

We used *scABC* and SCDE [18] to obtain cell type specific peaks for the experimental mixtures of GM12878/HEK293T and GM12878/HL-60 (cell types were defined based on *scABC* clustering results). SCDE is a Bayesian approach that identifies differentially expressed genes between two groups of samples only (here, we replace genes with peaks). Therefore we did not consider the *in silico* mixture of six cell types. To evaluate the accuracy of cell type specific peaks, we relied on the bulk DNase-seq from the ENCODE project (www.encodeproject.org) with accession numbers ENCSR000EMT, ENCSR000EJR, and ENCSR000ENU for GM12878, HEK293T, and HL-60, respectively. In particular, chromatin regions that are differentially accessible between GM12878 and HEK293T (also GM12878 and HL-60) bulk samples were treated as the gold standard. To determine these regions, we were not able to take advantage of existing methods such as DESeq2 [19] due to the lack of biological replicates for the DNase-seq samples. Instead, we applied the following heuristic method. Given a peak with length L, we define R as the number of read counts mapped to the peak. We also define R_0 as the number of read counts within 500,000 base pairs upstream and downstream from the peak center (background). We then calculate the bulk accessibility of the peak as

$$\frac{R}{R_0}\frac{1000,000}{L},$$

which is read counts fold change between the peak and background, normalized by their lengths. We assume that *scABC* (or SCDE) correctly identifies a peak, for instance, specific to HEK293T in the GM12878/HEK293T mixture, if the peak has i) the bulk accessibility > 2 in HEK293T and ii)

$$\frac{\text{bulk accessibility in HEK293T}}{\text{bulk accessibility in GM12878}} > T$$

We set T to 2 and 3 in Supplementary Figures 17 and 18, respectively. Condition i examines if the peak is open in the bulk data and condition ii tests if the open peak is differential in the mixture.

Applying the above conditions, we found that *scABC* calculated cell type specific peaks have 10%-60% higher overlaps with bulk differentially open regions, compared to SCDE (Supplementary Figs. 17 and 18). In addition, the top cell type specific peaks identified by *scABC* (i.e. the smallest p-values) are almost distinct across the cell types (Supplementary Figs. 10 and 11) while SCDE's top peaks (i.e. the largest/smallest z-scores) are not well separated, especially in the GM12878/HL-60 mixture (Supplementary Fig. 19).

3.8 The evolution of acute myeloid leukemia

To examine the capability of *scABC* in characterizing cancer progression, we drew our attention to leukemic evolution. Corces et al. [7] performed scATAC-seq on LSC and blast cells from two patient with leukemia as well as scATAC-seq on healthy LMPP and monocyte cells. In that study, bulk ATAC-seq data across myeloid developmental stages was obtained to build principle components (PC) that represent myeloid progression. Single cells were then projected onto myeliod PCs to determine the relationship between leukemia cells and myelopoietic differentiation.

We used *scABC* to cluster the mixture of LSC, blast, LMPP and monocyte cells without the use of bulk data. The sequencing depth of these cells is low (median: 13,937 unique mapped reads) compared to the previously analyzed mixture of six lines (median: 65,186 unique mapped reads), presenting significant computational challenges (Supplementary Fig. 1). As suggested by previous studies [20, 21], we hypothesized that distal regulatory

elements better capture blood cell identities. Hence, we collected predicted human enhancers from FANTOM5 (`http://fantom.gsc.riken.jp/5/`), ENCODE [22], and the Yue Lab (`http://promoter.bx.psu.edu/ENCODE/download.html`) and filtered out peaks that are not placed within the enhancers, leading to 7,602 peaks. We applied *scABC* to the total mixture of blood cells, resulting in $K = 2$ clusters (Supplementary Fig. 3). Supplementary Table 5 shows the clustering results and Supplementary Figure 13 depicts landmarks separability, heat map of cluster specific peaks, and chromVAR results. We observed that LMPPs and monocytes are clustered separately with the majority of blasts coupled with monocytes and LSCs mostly associated with LMPP. Furthermore, chromVAR identifies cluster specific active transcription factors through motif enrichment analysis. Such clear separation was not achievable when we did not incorporate enhancers into our analysis.

We also suspected that intermediate stages between LMPP and monocyte might be missing and thus, increased the number of cluster to four. As demonstrated in Supplementary Table 6 and Figure 14, one cluster dominated by LSCs and blasts might correspond to intermediate stages while others reflect LMPP and monocyte stages. In both scenarios, our conclusion is consistent with Corces et al. [7].

Supplementary References

[1] Cusanovich, D. A. *et al.* Multiplex single-cell profiling of chromatin accessibility by combinatorial cellular indexing. *Science* **348**, 910–914 (2015).

[2] Buenrostro, J. D. *et al.* Single-cell chromatin accessibility reveals principles of regulatory variation. *Nature* **523**, 486–490 (2015).

[3] Tay, S. *et al.* Single-cell NF-κB dynamics reveal digital activation and analogue information processing. *Nature* **466**, 267–271 (2010).

[4] Steidl, U. *et al.* Essential role of jun family transcription factors in PU. 1 knockdown–induced leukemic stem cells. *Nature Genetics* **38**, 1269–1277 (2006).

[5] Zhang, D. *et al.* Identification of potential target genes for rfx4_v3, a transcription factor critical for brain development. *Journal of neurochemistry* **98**, 860–875 (2006).

[6] Lun, A. T. & Smyth, G. K. From reads to regions: a Bioconductor workflow to detect differential binding in ChIP-seq data. *F1000Research* **4** (2015).

[7] Corces, M. R. *et al.* Lineage-specific and single-cell chromatin accessibility charts human hematopoiesis and leukemia evolution. *Nature Genetics* **48**, 1193–1203 (2016).

[8] Zhang, Y. *et al.* Model-based analysis of ChIP-Seq (MACS). *Genome Biology* **9**, R137 (2008).

[9] Dong, X. *et al.* Accurate identification of single-nucleotide variants in whole-genome-amplified single cells. *Nature Methods* **14**, 491–493 (2017).

[10] Dobin, A. *et al.* STAR: ultrafast universal RNA-seq aligner. *Bioinformatics* **29**, 15–21 (2013).

[11] Anders, S., Pyl, P. T. & Huber, W. HTSeq–a Python framework to work with high-throughput sequencing data. *Bioinformatics* **31**, 166–169 (2015).

[12] Lun, A. T., Bach, K. & Marioni, J. C. Pooling across cells to normalize single-cell RNA sequencing data with many zero counts. *Genome Biology* **17**, 75 (2016).

[13] Tibshirani, R., Walther, G. & Hastie, T. Estimating the number of clusters in a data set via the gap statistic. *Journal of the Royal Statistical Society: Series B (Statistical Methodology)* **63**, 411–423 (2001).

[14] Agresti, A. & Kateri, M. *Categorical data analysis* (Springer, Heidelberg, Berlin, Germany, 2011).

[15] Park, M. Y. *Generalized linear models with regularization*. Ph.D. thesis, Stanford University (2006).

[16] Friedman, J., Hastie, T. & Tibshirani, R. Regularization paths for generalized linear models via coordinate descent. *Journal of Statistical Software* **33**, 1–22 (2010).

[17] Cule, E., Vineis, P. & De Iorio, M. Significance testing in ridge regression for genetic data. *BMC Bioinformatics* **12**, 372 (2011).

[18] Kharchenko, P. V., Silberstein, L. & Scadden, D. T. Bayesian approach to single-cell differential expression analysis. *Nature Methods* **11**, 740–742 (2014).

[19] Love, M. I., Huber, W. & Anders, S. Moderated estimation of fold change and dispersion for RNA-seq data with DESeq2. *Genome Biology* **15**, 550 (2014).

[20] Heinz, S. *et al.* Simple combinations of lineage-determining transcription factors prime cis-regulatory elements required for macrophage and B cell identities. *Molecular Cell* **38**, 576–589 (2010).

[21] Xi, H. *et al.* Identification and characterization of cell type–specific and ubiquitous chromatin regulatory structures in the human genome. *PLoS Genetics* **3**, e136 (2007).

[22] Consortium, E. P. *et al.* An integrated encyclopedia of DNA elements in the human genome. *Nature* **489**, 57 (2012).
